# Identification of outliers in a genomic scan for selection along environmental gradients in the bamboo locust, *Ceracris kiangsu*

**DOI:** 10.1038/srep13758

**Published:** 2015-09-03

**Authors:** Xiao-Jing Feng, Guo-Fang Jiang, Zhou Fan

**Affiliations:** 1 Jiangsu Key Laboratory for Biodiversity and Biotechnology, College of Life Sciences, Nanjing Normal University, 1 Wenyuan Road, Nanjing, Jiangsu 210023, China

## Abstract

Identification of loci under divergent selection is a key step in understanding the evolutionary process because those loci are responsible for the genetic variations that affect fitness in different environments. Understanding how environmental forces give rise to adaptive genetic variation is a challenge in pest control. Here, we performed an amplified fragment length polymorphism (AFLP) genome scan in populations of the bamboo locust, *Ceracris kiangsu*, to search for candidate loci that are influenced by selection along an environmental gradient in southern China. In outlier locus detection, loci that demonstrate significantly higher or lower among-population genetic differentiation than expected under neutrality are identified as outliers. We used several outlier detection methods to study the features of *C. kiangsu*, including method DFDIST, BayeScan, and logistic regression. A total of 97 outlier loci were detected in the *C. kiangsu* genome with very high statistical supports. Moreover, the results suggested that divergent selection arising from environmental variation has been driven by differences in temperature, precipitation, humidity and sunshine. These findings illustrate that divergent selection and potential local adaptation are prevalent in locusts despite seemingly high levels of gene flow. Thus, we propose that native environments in each population may induce divergent natural selection.

Various environmental conditions, including distinctive latitude, may result in different physiological challenges, which in turn lead to morphological and molecular adaptations to local conditions^1^. Evidence from population genetics indicates that divergence evolution generally occurs in the presence of gene flow^2^, and it is well accepted that differentiation among populations can occur in the face of gene flow if adaptively driven^3^, and divergent selection may result in local adaptation and reduced gene flow between populations. Moreover, populations in different environments will initially genetically differ at a few key sites in their genomes, and the surrounding DNA may differ due to linkage disequilibrium. Uncovering the genetic basis of important adaptive traits in natural populations is a major goal to better understand how populations adaptively diverge in heterogeneous environments^4^. Recent studies have examined the number and location of genes involved in adaptation and evolution, and it has been suggested that genotypes caused by environment interactions allow for populations to evolve traits in their local habitat. This process and the resulting patterns are termed “local adaptation”^5^. Habitat fragmentation may weaken the connection between populations (isolation by distance) and lead to genetic divergence between populations. Differential adaptation or natural selection can then result in large allele frequency differences at loci between populations that control the involved traits, and these differences occur at a small number of DNA sites, but are potentially identifiable because linkage leads to ‘islands’ of differentiation around the selected sites, and a marker sampled within an ‘island’ will also be distinct. In particular, methods for genotyping large populations for many markers, including single nucleotide polymorphisms (SNPs), amplified fragment length polymorphisms (AFLPs), comparative anchor tagged sequences (CATs), and Expressed Sequence Tags (ESTs), have been developed. Although SNPs have been widely used to identify genome-wide loci by environment associations in model organisms^6^, we concentrated on the utility of AFLP markers because they can be easily applied to non-model organisms and used to generate hundreds of potential loci widely distributed across the genome^7^. AFLPs provide a quick and low-cost means of obtaining allele frequency data for large sample sizes and organisms for which little prior genetic knowledge is available^8^.

The detection of natural selection signatures within a genome allows for the understanding of what proportion of a genome or which genes are under the influence of natural selection. Genomic regions under selection are generally functionally important; hence, inferences regarding selection may provide useful information for identifying important genes^5^. Population genetics relies on the principle that all genomic loci are influenced by genome-wide evolutionary forces (genetic drift, gene flow), whereas locus-specific forces, such as selection, imprint a particular variability pattern on select loci. By comparing the genetic diversity of loci across the genome, it is possible to identify loci that have an atypical variation pattern (outlier loci), which are likely to be affected by selection. Strategies using population genomics are free from any prior knowledge about selectively advantageous genes or phenotypes and do not focus on a few loci but examine the effect of selection over the entire genome^9^,^10^. Outlier locus detection is a population-level analysis that uses estimates of population genetic differentiation (e.g., *F*_ST_). In outlier locus detection, loci with significantly higher or lower genetic differentiation than expected under neutrality are identified as outliers and are thus considered to possibly be under selection. Although a large number of markers are usually surveyed in the method, less than 5% are generally identified as outliers^11^.

The main drawback of the above methods is that they seldom link outlier loci with specific selection pressures (e.g., environmental) because it is notoriously difficult to determine genetic mechanism from the environmental effects on phenotypes. For adaptive divergence of populations to occur, the evolutionary force of directional selection must be stronger than the homogenizing effect of migration among populations and random genetic drift^12^. Spatial and temporal changes are heterogeneous in the natural environment, so divergent selection across natural environments can induce adaptive divergence resulting in local adaptation^12^,^13^. In addition to environmental variation, phylogeographic history, gene flow and population demographic processes all contribute to spatially structured genetic variation. Here, we examined genetic variation from an environmental angle to complement results from population genetic models. We applied the recently developed Samβada^14^ method to detect signatures of natural selection in locusts genotyped with AFLP markers. The idea behind this individual-based method is to correlate marker occurrence with environmental data in an allele distribution model, which uses geo-referenced environmental data and geo-referenced individual molecular genetic data. Molecular marker detection adaptive relevance relates the presence/absence of alleles to environmental data. It thus provides direct evidence to which ecological factor acts as a selective force. Over the last two decades, Samβada has been utilized in analyses of a wide variety of ecological patterns, including goat breeds^15^, ocellated lizards^4^ and gobiid fishes^16^.

Genome scans used in parallel with environmental data provide distinct clues for selective forces that act on molecular markers of adaptive relevance in the real landscape^17^ and will complement and strengthen robustness of the final set of loci identified as potentially under selection^18^. It is now possible to implement such an approach relatively cheaply on a genome-wide scale.

The *Ceracris kiangsu* bamboo locust is an important forest pest in China, and it is widely distributed throughout southern China^19^. One distinct characteristic of the species is its greater flight ability, presumably leading to frequent gene flows between populations. Fan *et al.* previously reported that this species has low levels of genetic structure and relatively high gene flow^17^, suggesting shallow evolutionary trajectories and limited or absent adaptive divergence among local populations.

In this study, we conducted an AFLP genome scan in *C. kiangsu* bamboo locust populations to identify candidate loci influenced by selection along an environmental gradient in southern China. Our objectives were to (1) test whether *C. kiangsu* populations adapted to local environmental conditions due to adaptive divergence and thus now display genomic signatures of divergent selection and (2) determine the environmental factors involved in local adaptation by explorative landscape genetic analysis.

## Results

### AFLP analysis

Four different primer combinations allowed us to amplify 360 AFLP bands, of which the mean number of fragments per individual was 81.8. The number of segregating fragments was 310, which accounted for 86.1% of the total fragments. We obtained 224 polymorphic markers.

### Outlier detection

**Figure 1:**
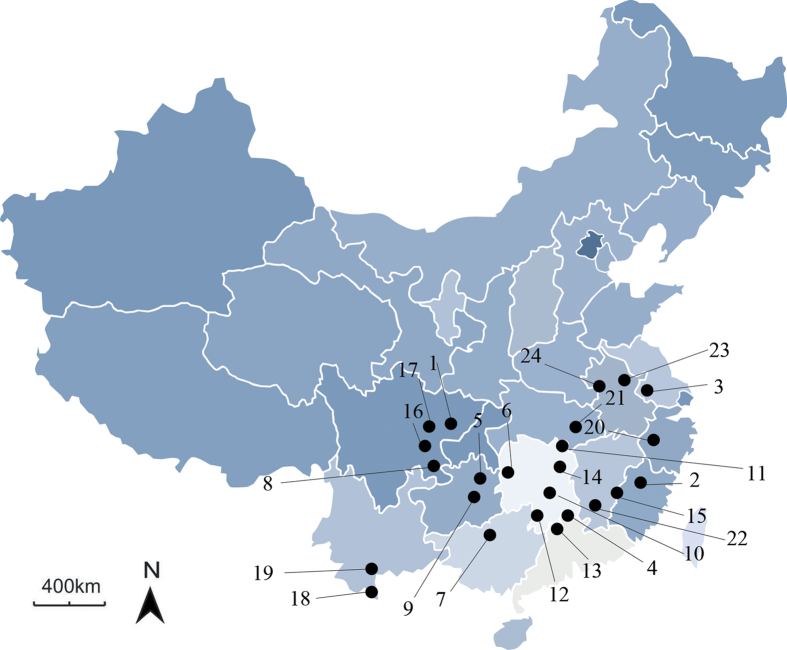
Map of the 24 sample localities for the bamboo locust with complete data. Each number beside black dots represents a sample locality respectively. Details for each site can be found in Table 1. Outline of China was downloaded from National Administration of Surveying, Mapping and Geoinformation (http://en.nasg.gov.cn/) for free and locations were produced using the software Adobe photoshop CS5.

We successfully tested a total of 224 polymorphic AFLP markers in 24 *C. kiangsu* populations across all sample sites in southern China (Fig. 1; Table 1). We performed all three outlier detecting methods with the same data set for global analysis.

**Figure 2:**
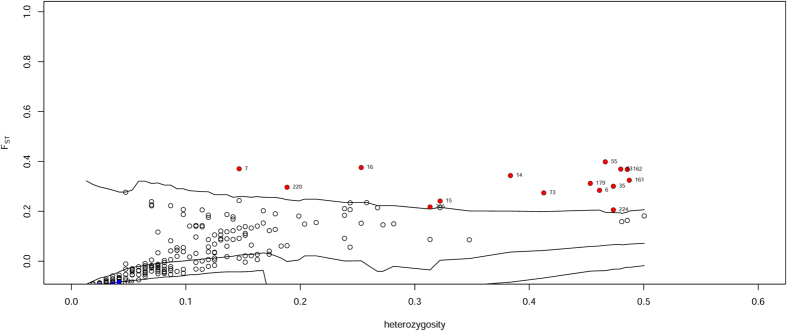
Distribution of FST values as a function of heterozygosity for interpopulational comparisons. Each dot represent an AFLP marker. The red dots above the upper line are classified as outliers potentially under divergent selection.

In DFDIST, the power for detecting differentiated outlier was high because of the low overall *F*_ST_ across sites^20^. This method identified a total of 16 outliers (Fig. 2). Among these outliers, one outlier presented a lower *F*_ST_ value than expected under neutrality, which suggests that it has potentially undergone balancing selection; the other 15 outliers presented higher *F*_ST_ values than expected under neutrality, corresponding to loci potentially influenced by directional selection.

**Figure 3:**
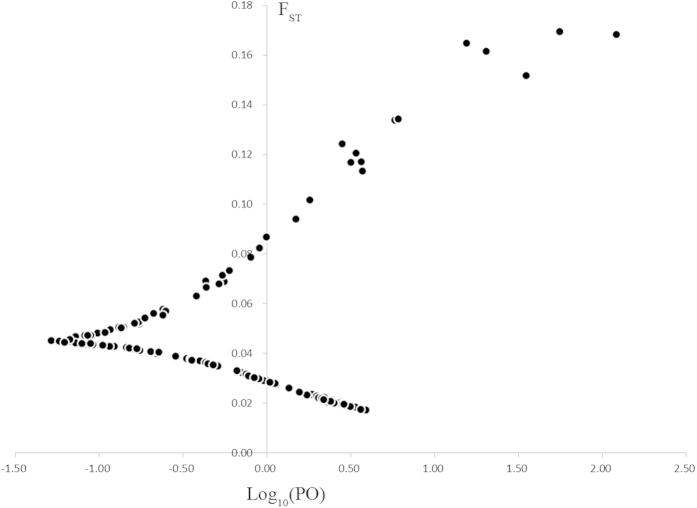
BayeScan 2.0 plot of 224 polymorphic amplified fragment length polymorphisms markers in global enhanced genome scan analysis of 393 individuals from the *C kiangsu* populations from China. *F*_ST_ is plotted against the log10 of the posterior odds (PO). The vertical line shows the critical PO used for identifying outlier markers. The 15 markers on the right side of the vertical line are candidates for being under positive selection.

In the BayeScan program, we detected 15 polymorphic loci with statistically significant patterns of divergent genetic differentiation (Fig. 3). Bayes factor identified high differentiation outliers at a threshold of PO >10. Among these, 13 loci had log 10 values above 1.5 (particularly strong) and ten had a log 10 Bayes factor of 1000, which corresponds to a posterior probability of one. Outliers detected by BayeScan were all considered candidate loci potentially under divergent selection.

Using Samβada, we identified 83 loci that significantly correlated to environmental variables following Bonferroni correction for both the Wald and G tests. Many loci were associated with more than one environmental variable. Among these loci, DFDIST consistently detected five loci : 6, 35, 55, 73 and 224. Though both Samβada and BayeScan detected 11 loci, BayeScan may be more effective in detecting outliers.

**Figure 4:**
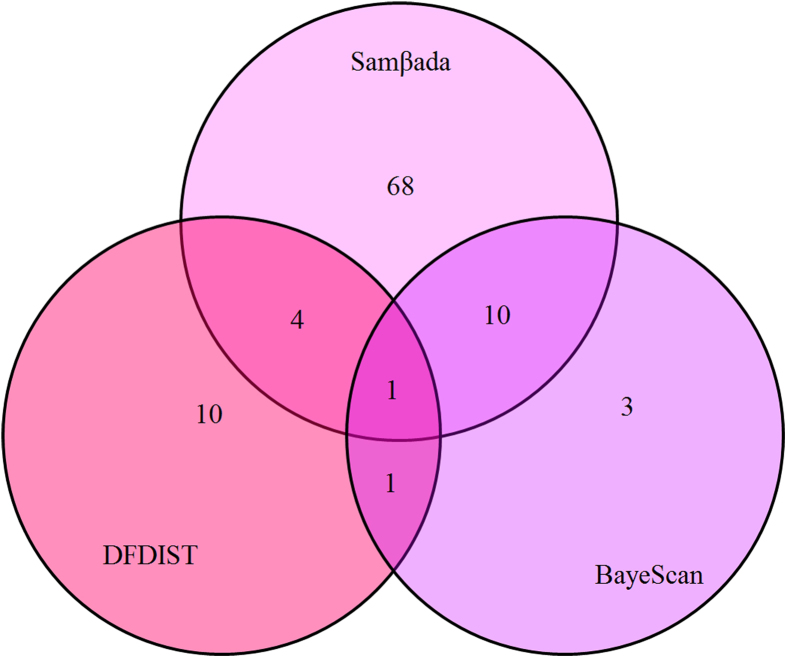
Venn diagrams illustrating the overlap of outliers in outlier detection for three different outlier detection methods.

The three methods identified a total of 97 outliers. Samβada identified the most outliers, up to 83 loci. DFDIST and BayeScan detected 29 candidate loci, which are demonstrated in Fig. 3. Among the 29 loci, DFDIST detected 17 loci and BayeScan detected 15 loci. The two programs identified two loci. BayeScan and Samβada both detected 11 loci, which are likely due to population divergence (Fig. 4).

### Association with environmental variables

We used in Samβada to test AFLP marker frequency variation in bamboo locusts in China for the environmental variables of annual sunshine (Sun), latitude (Lat), annual mean relative humidity (Hum), annual precipitation (Prec), annual mean temperature (T_mean_). We detected significant associations for 138 molecular markers and environmental variables out of 1120 combinations in wald score (p < 0.05). Samβada analysis results highlighted 14 outliers associated with annual sunshine (Sun), three outliers associated with latitude (Lat), thirteen outliers associated with annual relative humidity (Hum) and six with annual precipitation (Prec). Four loci showed an association with annual mean temperature (T_mean_). Loci 60 and 80 were associated with the most variables (each with four variables, Table 2), loci 8, 27, 30, 59, 80, 110, 126 and 224 associated with three variables and loci 35 and 55 associated with only one variable. On average, the strongest and most associated environmental variable was latitude, followed by annual sunshine, annual mean relative humidity, annual mean temperature and annual precipitation.

### Comparison of DFDIST and BayeScan with Samβada analysis

As listed in Table 2, Samβada highlighted five outliers among the 16 outliers detected by DFDIST as being significantly associated with the environmental variables tested. However, Samβada identified 12 of the 15 outliers detected by BayeScan to be significantly associated with variables (Table 2). Only one locus (locus 224) highlighted by Samβada variables was simultaneously detected by DFDIST and BayeScan as an outlier. This locus was associated with three climate variables by Samβada analysis, most strongly with Sun, Prec and Hum.

## Discussion

Our study suggests that loci under divergent selection are on various geographical scales in *C. kiangsu* through AFLP genome scan. In addition, Samβada highlighted a significant proportion of those loci with statistical significance.

### Outlier detection

The proportion of outliers detected with DFDIST (7.6%) is close to the proportion of outliers obtained by BayeScan (6.7%), which is also similar to the proportion previously reported by Nosil *et al.* that AFLP genome scans using DFDIST generally identify a proportion of 5–10%^21^. However, these studies are not directly comparable because the study design and chosen confidence may vary, such as pairwise comparisons^22^, global analysis^23^ or both^21^. The number of loci detected by Samβada was significantly increased compared to the other two approaches, which suggests that Samβada is more sensitive.

In our study, we used three approaches to detect outliers in the same data sets, but it is not comparable to the posterior probabilities from BayeScan and P-values obtained from DFDIST. However, the outliers detected by the three approaches were limited, possibly reflecting discrepancies in their methodologies. The main difference between DFDIST and BayeScan is that the *F*_ST_ within-populations is variable in BayeScan, but it is assumed to be the same across all populations in the former program^4^. However, the test performed by Beaumont concluded that gene flow between populations or isolation by distance did not have a strong effect. Nevertheless, previous studies have reported that neglecting population structure may produce high rates of false positives^24^. Some studies have adopted multiple pairwise comparisons among populations^25^ because such comparisons are more appropriate, which may diminish problems caused by unknown complexity and strengthen confidence for candidate loci.

Overall, DFDIST appears to be more sensitive than BayeScan (DFDIST: 7.6%, BayeScan: 6.7%). Previous studies have indicated that BayeScan usually detects a high percentage of true selective loci and less than 1% of outliers (false positives) under a fully neutral model. The percentage of outliers detected by this software always correlates with the true percentage of selective loci in the genome^26^. The Bayesian method assumes that gene frequencies under any neutrally structured population model can be approximated by a multinomial Dirichlet distribution^10^. However, Dirichlet distribution would not hold if different samples were drawn from the same population or if sampled populations shared more recent ancestry than others^27^. In addition, Lotterhos^27^ emphasized that the default settings in BayeScan may result in many false positives that suggest balancing selection. It suggests that some *F*_ST_ outliers may be false positives. Although Pérez-Figueroa *et al.* compared DFDIST, DETSELD and BayeScan and found that BayeScan was more efficient under a wide range of scenarios^26^, a recent simulation study compared FDIST2, BayeScan, and two recent methods (FLK and Bayenv2), and showed that the default settings in FDIST2 and BayeScan led to many false positives, suggesting balancing selection^27^.

Samβada is clearly more sensitive for detecting several times loci than DFDIST and BayeScan. However, the eleven loci consistently detected by both Samβada and BayeScan were highly supported by statistics.

Indeed, there are some limitations of genome scans, such as sensitivity to phylogeographic structure and bottlenecks^28^. Four loci had log10 values as high as 5, which was the value ascribed to posterior probabilities of 1 (Bayes factor is infinity). Accordingly, the majority of these outliers are likely affected by directional selection and not simply by random chance. Our evidence for selection is as strong as possibly achievable given the statistical method and the number of loci available. The number of outliers that we detected for the data set is below the range found in other studies^29^ (5–10%). The high proportion of outliers in previous studies likely represents a high rate of false positives. False positives are also possible among our candidate loci and could represent stochastic processes or linkage to other candidates^30^. Therefore, outliers in *C. kiangsu* require further study and detection by newly developed methods.

When applying Samβada analysis to the AFLP data set, some loci identified as outliers in Samβada associated with environmental variables were also detected by DFDIST or BayeScan (Table 2). Samβada is a useful method when simultaneously searching for linkages within many climate variables, which provides insight into which selective forces may be in play. Because many climate variables are inter-related, information on environmentally related requirements of a species is important to determine which environmental variables are the most critical forces. Additionally, the variables tested must be ecologically relevant instead of random changes within the genetic data^4^. Samβada is a new analysis approach that can better identify associations with environmental variables.

### Association with environmental variables

Local directional selection appears to be general and relatively widespread and can be found on a number of geographical scales, as the global or regional outliers were not exclusively dependent on one or a few particularly divergent populations. Although it is difficult to completely disentangle the effects of geographic and environmental distance, a higher proportion of identified outlier loci were associated with environmental parameters and geographic variables. These data suggest that environmental factors are potentially responsible for adaptive divergence among populations^31^.

The results from Samβada analysis for *C. kiangsu* revealed possible evolutionary forces of outlier loci along the environmental gradient in China. The strongest associations selected for latitude (Lat). Notably, latitude is an easily accessible ecological factor, but it is also a compound factor because temperate regions, temperature, humidity, sunshine and precipitation all change with latitude.

All 31 associations were significantly correlated with annual sunlight. It is well known that sunlight is the primary energy source in ecosystems and a particularly important factor for insects. This locust generally prefers areas with relatively little sunlight because the intensity and duration of the sunlight affect its movement and feeding abilities and the local bamboo species’ development^32^.

Alternatively, because temperature is important for proper protein and physiological process function, we identified a total of 23 associations with annual mean temperature. Temperature differences among geographical locations could alone drive the evolutionary response for all genes in concert. Our landscape genetic analysis partially explains this hypothesis, as almost all outlier loci significantly associated with temperature. For example, a phenomenon was previously observed that grasshoppers were negatively affected by fall temperatures in the winter, which may affect embryonic development before hatching and diapause^33^. Furthermore, in the *Sigaus australis* grasshopper, temperature may affect water loss, body size and population dynamics^34^.

Altogether, we identified 19 associations with annual precipitation, which may be considered the least important variable. This variable probably does not influence locusts’ activity to the same extent as latitude and sunshine. However, due to precipitation decreases from south to north in China, annual precipitation can reach values as high as 1800 mm in Guilin, but only 800 mm in Ziyang. Precipitation influences grasshoppers in many aspects. For example, the population density is lower in regions with little precipitation^35^, and increased precipitation may affect the occurrence of the late-season grasshopper^36^. Franzke previously suggested that climate change events, such as drought and heavy rain, are likely to affect plants and influence the performance of population dynamics in herbivorous insects. Drought events may increase population performance (development time, body size, mortality, growth rate) in grasshopper and moisture may cause negative population trends^37^. Another study proposed that summer rainfall may positively affect plants, hence affecting the quantity and quality of forage production and grasshopper populations^33^.

We identified another 28 associations with annual relative humidity. *C. kiangsu* requires a large amount of moisture from the nymph stage to the adult stage, and a previous study has shown that humidity correlates to *C. kiangsu* movement and feeding abilities^32^. The combination of environmental variables may lead to a comprehensive influence on locusts. For example, Joachim *et al.* (2004) suggested that sunlight and temperature and some other ecologically factors may affect the number of juvenile instars and morality in nymphs^38^.

### Future directions

Our study identified a list of loci potentially under the influence of selection in bamboo locust. The loci need to be isolated and sequenced, and sequenced fragments must be mapped to the outlier fragments within the genome. If the outlier’s sequence is not homologous with any known gene, it may be belong to an unknown regulatory region or a non-coding fragment linked with the selection target^4^. The determination of outlier function and characterization is necessary to identify their involvement in local adaptation of *C. kiangsu* and the effects of environmental variables onto the molecular mechanism. However, mapping of loci to known genomic sequences requires the availability of detailed genomic information of closely related species. Combined studies on adaptive and neutral molecular markers will largely contribute to our understanding of genetic differentiation among *C. kiangsu* populations and will allow us to investigate the ‘migration of adaptation’^28^. Our study also sheds light on the use of genome scan methods to identify evolutionary pressures on candidate loci in a local population. Although public databases offer a good source of sample coordinates and environmental information, more ecologically relevant and detailed information, such as the maximum and lowest temperature, host-plant species and abundance of food sources and genetic information, require further collection. Compiling information on phenotypic, ecological and genomic data may be fruitful to investigate species adaptation.

## Methods

### Sample collection, DNA extraction and AFLPs

A total of 393 *C. kiangsu* individuals were collected in the field from 24 locations in China, which covered all of the species’ distributions over China ranging from 2007 to 2012 (Fig. 1; Table 1). A total of 24 populations were used for subsequently analysis. Locusts were collected using a sweep net and subsequently preserved in absolute ethanol. Genomic DNA was extracted from femurs using a Wizard^®^ Genomic DNA Purification Kit (Promega, Madison, WI, USA) according to the manufacturer’s instructions and stored at −30 °C until needed. All DNA extracts for AFLP were run on 1% agarose gels, and samples that did not have high concentrations of high molecular weight DNA or that appeared excessively sheared were excluded from AFLP analysis^20^. Sample sizes for AFLP analysis ranged from two to ten.

The original AFLP protocol of Vos *et al.* was applied with a few modifications^39^. Individuals with high consistency and purity quotients of genomic DNA were used. Because grasshopper species have a larger genome than many other insects, a longer enzyme digestion was performed to obtain more polymorphic fragments. A total of 400 ng genomic DNA was digested at 37 °C with 0.2 μL *Eco*RI and 0.5 μL *Mse*I (both Fermentas, with 2 × Buffer R) for 3 h followed by 70 °C for 15 min to ensure enzyme inactivation. *Eco*RI/*Mse*I adapters were ligated to the digested product using T4-DNA-Ligase (FERMENTAS) at 20 °C overnight. A total of 16 primer combinations containing one selective base were used. Preselective amplification was performed in a total volume of 30 μL including 3 μL diluted restriction-ligation DNA, 3 μL 10 × buffer, 2.4 μL MgCl_2_, 1.6 μL dNTPs, 0.4 μL rTaq (TAKARA) and 1 μL *Eco*RI + N primer and 1 μL *Mse*I + N primer. The thermal cycling parameters for preselective amplification were as follows: 2 min at 94 °C, 30 cycles of 30 sec at 94 °C, 1 min at 56 °C, 1 min at 72 °C, followed by 10 min at 72 °C. Ligations were diluted 1:20, and 2 μL of the diluted preselective amplification product was used in selective amplification. Selective amplification was performed in a total volume of 20 μL with 2 μL diluted preselective amplification product, 2 μL 10 × buffer, 1.6 μL MgCl_2_, 1.6 μL dNTPs, 0.6 μL rTaq (TAKARA) and 1 μL *Eco*RI + 3 primer (labeled with FAM) and 1 μL *Mse*I primer. Four primer combinations (E–AGG/M–CAG, E–AGG/M–CTT, E–AGC/M–CTC, E–AAG/M–CAG, where E is *Eco*RI, M is *Mse*I) were used in this step. Selective amplification products were visually measured on an ABI 3700 DNA analyzer (Shanghai Sangon Biotech Co., Ltd.).

Fragment data were analyzed with GeneMarker version 2.20 (Demo). Fragments of size 50–500 bp were scored as present or absent. Minimum fragment signal intensity was initially used for all fragments. The signal intensity was measured as relative fluorescent units (RFU) of 500 or 1000 depending on the primer set^20^.

### Environmental data

To test the effect of environment on genetic diversity, environmental data were required at all sampling locations using the geographical coordinates where locusts were sampled. Climate data were obtained from a public website. The annual sunshine (Sun), annual relative humidity (Hum), annual precipitation (Prec), and annual mean temperature (T_mean_) from 2000 to 2012 were provided by the statistical bureau (http://www.stats.gov.cn/). The data for latitude, longitude and elevation (Table 1) were obtained from Google Earth (http://www.google.com/earth/download/ge/agree.html).

### Outlier detection

Fragmentized information was exported as 0/1 popmatrix by GeneMarker and transformed in format in the AFLPDAT program^40^. To identify candidate loci potentially influenced by selection among sites across China, two different *F*_ST_ outlier detection approaches were performed: DFDIST^41^ and BayeScan^42^. DFDIST (http://www.rubic.rdg.ac.uk/~mab/stuff/) is a modification of distributed FDIST^41^ and FDIST2^43^, and an infile of DFDIST was created by the AFLP convert program. DFDIST calculates the simulated values for heterozygosity an *F*_ST_ using Zhivotovsky’s approach^44^. DFDIST estimates the probability that a locus may be under selection by observed *F*_ST_ and *H*_E_ compared to simulated neutral distributions. DFDIST calculated a “trimmed” mean *F*_ST_ value by removing 30% of the highest and lowest *F*_ST_ values using the null distribution, which is the neutral *F*_ST_ value. In the simulation loci, *F*_ST_ values above the upper 99% quantile were considered as being potentially under directional selection consistent with population difference.

The other method for detecting signatures of natural selection was implemented in BayeScan 2.0 (http://www.cmpg.unibe.ch/software/bayescan/) using the Bayesian likelihood method via reversible-jump Monte Carlo Markov chain (MCMC). Generally, such Bayesian approaches^42^,^43^ assume that allele frequencies within populations follow a Dirichlet distribution^45^,^46^,^47^. It directly estimates the probability that each locus is subject to selection using a Bayesian method. The method uses population-specific and locus-specific components of *F*_ST_ coefficients and assumes that allele frequencies follow a Dirichlet distribution. BayeScan considers all loci in its analysis and is robust when examining complex demographic scenarios for neutral genetic differentiation^42^. This enhanced Bayesian method directly infers the posterior probability of each locus to be under the effect of selection by defining and comparing two alternative models. One model includes the effect of selection (M1), while the other (M2) excludes it^42^. These posterior probabilities can then be used for model choice using posterior odds (PO), which is the ratio of posterior probabilities of the models and measures how much more likely model M1 (with selection) is compared to model M2 (without selection). When using the same prior for both models (M1 and M2), the posterior odds are reduced to the Bayes Factor. Jeffreys^48^ (1961) proposed a logarithmic scale for model choice defined as: >3 substantial (log_10_PO > 0.5); > 10 strong (log_10_PO > 1.0); >32 very strong (log_10_PO > 1.5); and >100 decisive evidence for accepting a model (log_10_PO > 2.0). In our genome scans, a threshold for PO > 10 (strong) was used as a marker to be considered under selection. This corresponds to a posterior probability greater than 0.91 for the model accounting for selection. For the Markov chain Monte Carlo algorithm implemented in BayeScan 2.0, 20 pilot runs of 2000 iterations were used to adjust the proposal distribution to have acceptance rates between 0.25 and 0.45 for the runs. Afterwards, a burn-in of 50,000 iterations followed by 50,000 iterations were used for estimation using a thinning interval of 10.

Thirdly, Samβada analysis was implemented, and this method was designed for amplified fragment length polymorphism (AFLP) data. The logistic regression model was performed such that individuals are coded with the presence/absence of an allele. The model fit to be was considered significant when both the G and Wald tests were significant following Bonferroni correction at a 99% confidence level.

### Association with environmental variables

Associations between markers and environmental variables were directly tested using an individual-based analysis that estimates spatial coincidence implemented in the Samβada program, available at lasig.epfl.ch/sambada. Here, individuals were coded with the presence/absence of an allele, and AFLP polymorphisms were visually scored as dominant markers, coded with 1 for the presence and with 0 for the absence of the band. Afterwards, associations between the allele and environmental parameters were tested across sites. The environmental variables implemented were annual sunshine (Sun), latitude (lat), annual relative humidity (Hum), annual precipitation (Prec) and annual mean temperature (T_mean_). The univariate output file consisted of each possible molecular and environmental variable combination, so a p-value was calculated for the wald scores test, where wald scores were compared to a chi-square distribution with 1 degree of freedom, which is the regression coefficient divided by its standard error and hence a Chi-square distributed with 1 degree of freedom. Those corrected p-values < 0.5 were considered highly significant associations between the marker and environmental variable.

## Additional Information

**How to cite this article**: Feng, X.-J. *et al.* Identification of outliers in a genomic scan for selection along environmental gradients in the bamboo locust, *Ceracris kiangsu*. *Sci. Rep.* **5**, 13758; doi: 10.1038/srep13758 (2015).

## Figures and Tables

**Table 1:** Sampling site, geographical coordinates, annual sunshine (Sun), latitude (Lat), mean annual relative humidity (Hum), annual precipitation (Prec), annual mean temperature (T_mean_), and sample size of sampled *C. kiangsu* populations.

No.	Sampling site	Lat	Longitude	T_mean_(°C)	Prec(mm)	Sun(h)	Hum	N
1	Jinyunshan, Chongqing	29°50′14″N	106°23′46″E	18.76	1065.60	1023.96	77.8	25
2	Jianou, Fujian	27°02′08″N	118°14′14″E	20.38	1534.38	1444.33	72.3	14
3	Nanjing, Jiangsu	24°29′18″N	117°21′01″E	16.53	1107.38	1887.74	70.0	2
4	Guangning, Guangdong	23°36′17″N	112°23′18″E	21.80	1581.68	1643.26	un	34
5	Guilin, Guangxi	25°18′25″N	110°23′40″E	19.52	1786.08	1466.80	un	10
6	Quanzhou, Guangxi	25°55′43″N	111°09′44″E	19.52	1786.08	un	un	20
7	Rongan, Guangxi	25°12′29″N	109°23′45″E	21.20	1439.48	un	un	18
8	Jinping, Guizhou	26°43′04″N	109°10′52″E	16.00	1059.30	1350.40	74.5	6
9	Mayanghe, Guizhou	28°41′47″N	108°16′16″E	17.20	1121.95	927.00	71.5	2
10	Hengyang, Hunan	27°07′01″N	112°41′36″E	19.05	1219.08	1571.65	un	4
11	Huarong, Hunan	29°35′23″N	112°32′17″E	18.38	1159.52	1769.97	un	20
12	Shuangpai, Hunan	26°06′30″N	111°49′28″E	18.73	1295.62	1437.55	un	18
13	Taoyuan, Hunan	28°54′09″N	111°29′20″E	18.45	1253.45	1585.60	un	46
14	Changsha, Hunan	28°10′11″N	112°40′06″E	18.41	1302.86	1681.94	72.2	19
15	Shicheng, Jiangxi	26°19′35″N	116°20′36″E	20.00	1242.95	1768.38	68.8	19
16	Changning, Sichuan	28°29′57″N	104°55′58″E	18.70	877.58	921.74	76.0	19
17	Ziyang, Sichuan	30°07′45″N	104°37′44″E	17.66	821.26	1256.52	77.0	5
18	Mengla, Yunnan	21°29′18″N	101°33′23″E	22.20	1225.95	un	un	5
19	Menglun, Yunnan	21°55′25″N	101°15′56″E	22.20	1225.95	un	un	7
20	Quzhou, Zhejiang	30°19′03″N	119°25′57″E	17.83	1568.21	1839.59	un	14
21	Wuhan, Hubei	31°05′30″N	114°21′01″E	17.67	1188.63	1806.45	71.6	20
22	Pingxiang, Jiangxi	27°37′22″N	113°51′15″E	18.50	1629.18	1559.65	77.8	31
23	Guangde, Anhui	30°47′19″N	119°28′51″E	un	un	un	un	15
24	Shucheng, Anhui	31°22′05″N	116°59′13″E	un	un	un	un	15

un: unknown data.

**Table 2:** List of the 29 outliers detected by DFDIST and BayeScan.

Outlier	DFDIST P-value	BayeScan posterior probability	Samβada
6	**0.000**	0.054	Hum, Sun
**7**	**0.000**	**1.000**	
8	0.858	**0.939**	Prec, Sun, Lat
13	0.970	**0.972**	
14	**0.000**	0.060	
15	**0.001**	0.071	
16	**0.000**	0.777	
27	0.535	**0.982**	Hum, Sun, Lat
30	0.312	**1.000**	Hum, Sun, T_mean_
35	**0.000**	0.057	Hum
55	**0.000**	0.057	Sun
59	0.810	**1.000**	Hum, T_mean_, Sun
60	0.922	**1.000**	T_mean_, Lat, Prec, Sun, Hum
63	0.781	**0.953**	
73	**0.000**	0.785	Hum, Sun
80	0.559	**0.082**	Hum, Sun, T_mean_, Prec
84	0.761	**1.000**	Hum Sun
91	0.784	**1.000**	Hum, Sun
93	**0.000**	0.104	
109	0.966	**1.000**	
110	0.875	**1.000**	Hum, Sun, Prec
123	**0.005**	0.642	
126	0.989	**0.992**	Hum, Sun, Prec
161	**0.000**	0.138	
162	**0.000**	0.059	
179	**0.000**	0.665	
205	**0.002**	0.784	
220	**0.001**	0.510	
224	**0.002**	**1.000**	Sun, Prec, Hum

For each outlier, values of posterior probability above 0.99 in bold are indicated. Numers of marker underlined are both detected by Samβada as outlier loci. Then the outliers were detected by spatial analysis method (Samβada), the climatic variables most strongly associated with locus are indicated.

Sun: annual sunshine, Lat: latitude, Hum: annual relative humidity, Prec: annual precipitation, T_mean_: annual mean temperature.
